# Multiple Metabolic Alterations Exist in Mutant PI3K Cancers, but Only Glucose Is Essential as a Nutrient Source

**DOI:** 10.1371/journal.pone.0045061

**Published:** 2012-09-13

**Authors:** Rebecca Foster, Sue Griffin, Suzanne Grooby, Ruth Feltell, Cindy Christopherson, Monica Chang, John Sninsky, Shirley Kwok, Chris Torrance

**Affiliations:** 1 Horizon Discovery Ltd, Cambridge, Cambridgeshire, United Kingdom; 2 Celera Corp, Alameda, California, United States of America; Duke University, United States of America

## Abstract

Targeting tumour metabolism is becoming a major new area of pharmaceutical endeavour. Consequently, a systematic search to define whether there are specific energy source dependencies in tumours, and how these might be dictated by upstream driving genetic mutations, is required. The PI3K-AKT-mTOR signalling pathway has a seminal role in regulating diverse cellular processes including cell proliferation and survival, but has also been associated with metabolic dysregulation. In this study, we sought to define how mutations within *PI3KCA* may affect the metabolic dependency of a cancer cell, using precisely engineered isogenic cell lines. Studies revealed gene expression signatures in *PIK3CA* mutant cells indicative of a consistent up-regulation of glycolysis. Interestingly, the genes up- and down-regulated varied between isogenic models suggesting that the primary node of regulation is not the same between models. Additional gene expression changes were also observed, suggesting that metabolic pathways other than glycolysis, such as glutaminolysis, were also affected. Nutrient dependency studies revealed that growth of *PIK3CA* mutant cells is highly dependent on glucose, whereas glutamine dependency is independent of *PIK3CA* status. In addition, the glucose dependency exhibited by *PIK3CA* mutant cells could not be overridden by supplementation with other nutrients. This specific dependence on glucose for growth was further illustrated by studies evaluating the effects of targeted disruption of the glycolytic pathway using siRNA and was also found to be present across a wider panel of cancer cell lines harbouring endogenous *PIK3CA* mutations. In conclusion, we have found that *PIK3CA* mutations lead to a shift towards a highly glycolytic phenotype, and that despite suggestions that cancer cells are adept at utilising alternative nutrient sources, *PIK3CA* mutant cells are not able to compensate for glucose withdrawal. Understanding the metabolic dependencies of *PIK3CA* mutant cancers will provide critical information for the design of effective therapies and tumour visualisation strategies.

## Introduction

The PI3K-AKT-mTOR pathway is a key oncogenic signalling pathway and as such has a central role in regulating cell proliferation, cell survival, cancer cell invasion and metastasis [1]–[3]. Hyper-activation of the pathway is common in human cancers and can be achieved in a number of ways, including mutation of *PIK3CA*. *PIK3CA*, the gene encoding the alpha catalytic subunit of the kinase, is mutated in approximately 15% of colorectal tumours and approximately 30% of breast cancers, with most mutations occurring at three hotspots, *E542K*, *E545K* and *H1047R* [4]–[9]. The first two hotspot mutations, *E542K* and *E545K*, are found in exon 9, which lies within the helical domain, while *H1047R*, the most prevalent mutation in breast cancer, is found in exon 20, which lies within the kinase domain. Mutations within both regions lead to constitutive activation of PI3K-AKT-mTOR signalling [3], [5], [10].

The PI3K-AKT-mTOR pathway not only regulates cell proliferation and cell survival, but is an important pathway in the control and regulation of cancer metabolism [2], [11]–[15]. The integration of metabolic changes with proliferation signalling is crucial to conferring a competitive advantage upon a cancer cell, resulting in oncogenesis and metastasis.

**Figure 1:**
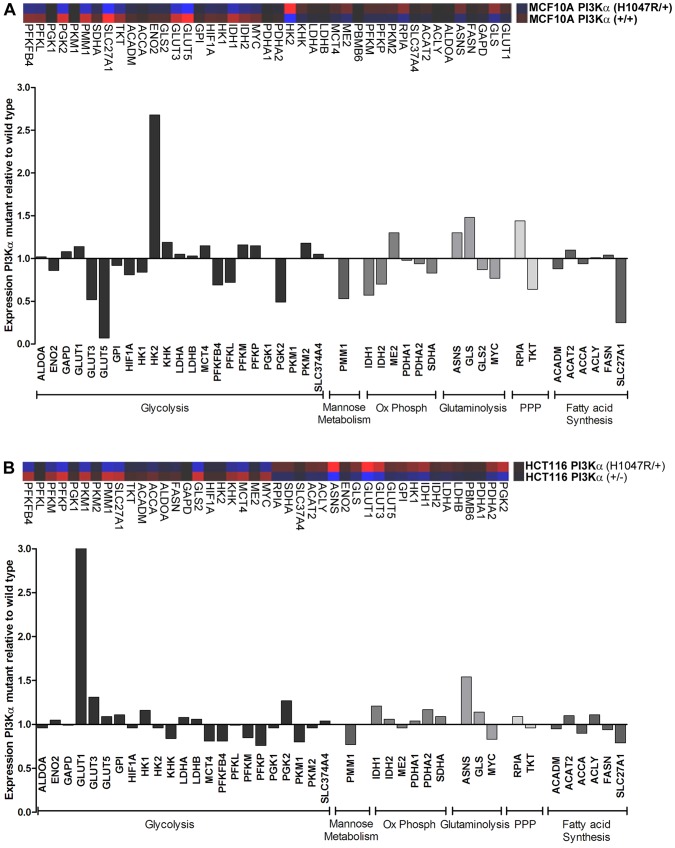
Metabolic gene expression profiles are altered as a result of the H1047R *PIK3CA* activating mutation. Comparative gene expression analysis was performed for a set of genes representing different metabolic pathways using (A) the MCF10A PI3Kα isogenic pair (+/+ and H1047R/+) and (B) the HCT116 PI3Kα isogenic pair (+/− and H1047R/+). Ox Phosph, oxidative phosphorytion; PPP, pentose phosphate pathway. Replicate data sets have been averaged and gene expression changes for PI3Kα mutant cells are represented as fold-change relative to PI3Kα wild type cells. Different glycolysis genes were up-regulated as a result of *PIK3CA* mutation in the different cell models.

Metabolic adaption of cancer cells is a well known phenomenon, with the studies by Otto Warburg that demonstrated cancer cells consume glucose at an elevated rate even in the presence of oxygen, remaining seminal to the field (reviewed [16]–[18]). Metabolic alterations do not simply result in changes in energy production, but are key for regulating the production of macromolecular building blocks required by a cell and for the maintenance of redox balance [16], [19]–[22]. Although aerobic glycolysis (the Warburg effect) is the most widely documented metabolic activity in tumours and cancer cell lines, additional pathways such as mitochondrial glutamine metabolism and fatty acid synthesis cooperate to produce the macromolecules needed to support continued cell growth. Indeed, glutamine contributes to many core metabolic tasks of proliferating tumour cells and is often only considered secondary to glucose in terms of importance in tumour cell metabolism. Glutamine is the most abundant amino acid in human plasma, and cancer cells metabolise glutamine in excess of any other amino acid [23]–[25]. Notwithstanding, a number of published studies have linked other amino acid dependencies with metabolic effects and survival of cancer cells [26]–[28]. These nutrient studies highlight the complex and somewhat flexible nature of tumour metabolism, since cancer cells can not only adapt and switch between different metabolic pathways, but also have simultaneous access to multiple nutrients.

The PI3K signalling pathway, largely through its effects on AKT and mTOR, has been associated with regulation of a number of metabolic effects, including glucose uptake, stimulation of the Warburg effect, and enhanced synthesis of lipids and proteins [11], [29], [30]. AKT activation for example has been shown to stimulate glycolysis by increasing expression and membrane translocation of the glucose transporter GLUT4. Active AKT also acts via GSK3β to regulate glycogen synthase and promote the association of Hexokinase 2 with the mitochondrial membrane, where Hexokinase 2 can more readily phosphorylate glucose [11], [13], [29]–[33].

**Figure 2:**
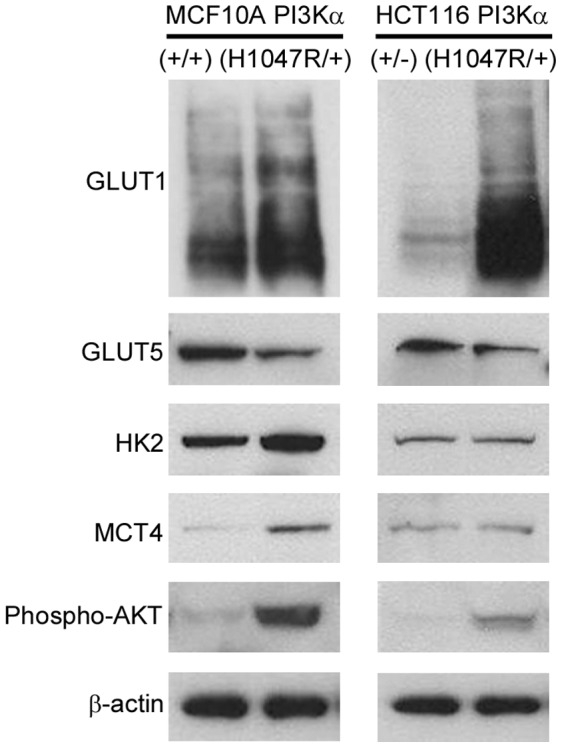
Key glycolysis and other metabolic associated proteins are up-regulated by *PIK3CA* mutation. Protein expression was evaluated by Western blotting to confirm gene expression data in both (A) the MCF10A PI3Kα isogenic pair (+/+ and H1047R/+) and (B) the HCT116 PI3Kα isogenic pair (+/− and H1047R/+) of cell lines.

While historical studies have demonstrated connections between the PI3K-AKT-mTOR pathway and metabolism, the model systems used have often relied on comparisons between cell lines that have multiple genetic differences or used over-expression strategies that are not reflective of patient tumour genetics. The predominant focus of research has centred on the influence of tumour genetics on glucose metabolism. However, genetic alterations can also dictate alternative nutrient and metabolic dependencies.

In order to explore how endogenous *PIK3CA* mutations specifically alter metabolic pathways and to better understand whether any of these potential changes establish therapeutically targetable cellular metabolic dependencies, we have performed focused metabolic gene expression analysis, nutrient switching and siRNA experiments using isogenic cell line models that are genetically identical, apart from the mutation status of the endogenous *PIK3CA* gene.

## Materials and Methods

### Cell Culture

All MCF10A and HCT116 X-MAN™ isogenic cell lines were obtained from Horizon Discovery Ltd (http://www.horizondiscovery.com). The following X-MAN™ isogenic cell lines were used in this study: MCF10A PI3Kα (H1047R/+), heterozygous knock-in of *PIK3CA* kinase domain activating mutation (HD 101–011); MCF10A PI3Kα (E545K/+), heterozygous knock-in of *PIK3CA* helical domain activating mutation (HD 101–002); HCT116 PI3Kα (+/−), knock-out of *PIK3CA* kinase domain mutant allele (*H1047R*) in heterozygous parental cells (HD 104–007). Parental cell lines, MCF10A PI3K (+/+) and HCT116 PI3K (H1047R/+) were also used. All isogenic cell lines were created using rAAV-mediated homologous recombination to precisely and stably alter specific target genomic loci [3], [34]. All MCF10A isogenic cell lines were maintained in DMEM/F12 media (PAA) supplemented with 5% horse serum (Invitrogen), 10 µg/ml insulin (Sigma), 0.5 µg/ml hydrocortisone (Sigma) and 0.1 µg/ml cholera toxin (Sigma). MCF10A parental cells were additionally supplemented with 20 ng/ml hEGF (R&D Systems). HCT116 isogenic cell lines were maintained in McCoy’s 5A media (Invitrogen) supplemented with 10% foetal bovine serum (PAA).

Non-isogenic cell lines harbouring *PIK3CA* mutations were purchased from ATCC (BT20, DLD-1, MDA-MB-453 and RKO) and ECACC (MCF7 and T47D), and maintained according to the supplier recommendations.

**Figure 3:**
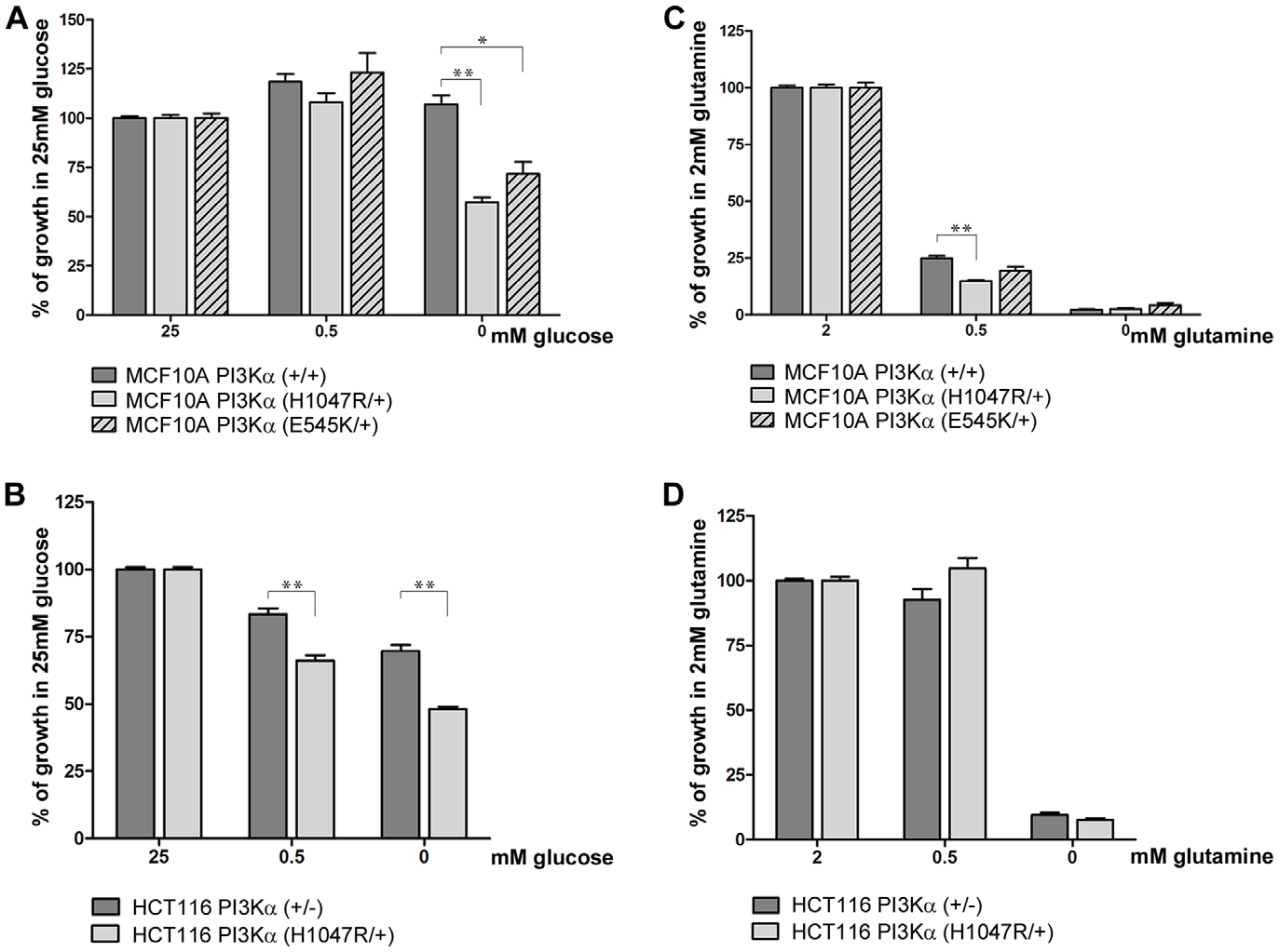
Growth of *PIK3CA* mutant cancers is dependent on the presence of glucose, but not glutamine. MCF10A PI3Kα isogenic cells (+/+, H1047R/+ and E545K/+) and HCT116 isogenic cells (+/− and H1047R/+) were grown in media containing 2 mM glutamine and the indicated concentrations of glucose (A and B respectively), or in media containing 25 mM glucose and the indicated concentrations of glutamine (C and D respectively) for 120 hours and cell growth assessed using SRB. Growth of each cell line is expressed relative to growth in 25 mM glucose or 2 mM glutamine (*p<0.05; **p<0.01).

**Figure 4:**
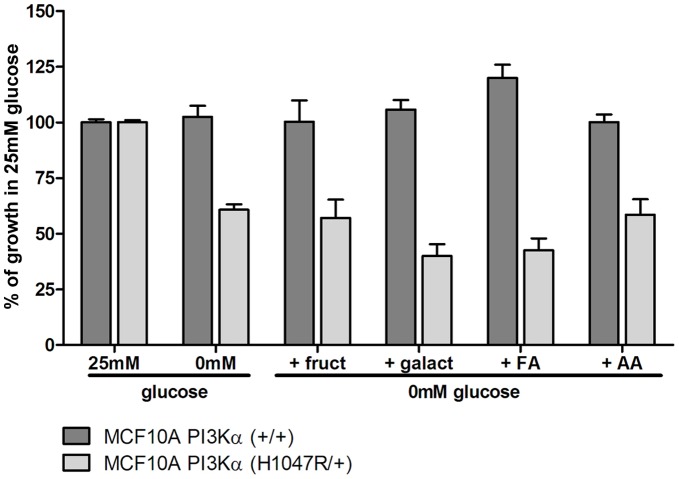
*PIK3CA* mutation specific glucose dependency cannot be overridden by nutrient supplementation. MCF10A PI3Kα (+/+) and MCF10A PI3Kα (H1047R/+) cells were grown for 120 hours in media containing 25 mM glucose or without glucose as indicated. All media contained 2 mM glutamine. Additionally, cells grown without glucose were supplemented with either 0.5 mM fructose (+ fruct), 10 mM galactose (+ galact), fatty acid cell culture supplement (+FA) or 0.1 mM aspartic acid (+AA). Cell growth was assessed using SRB.

**Figure 5:**
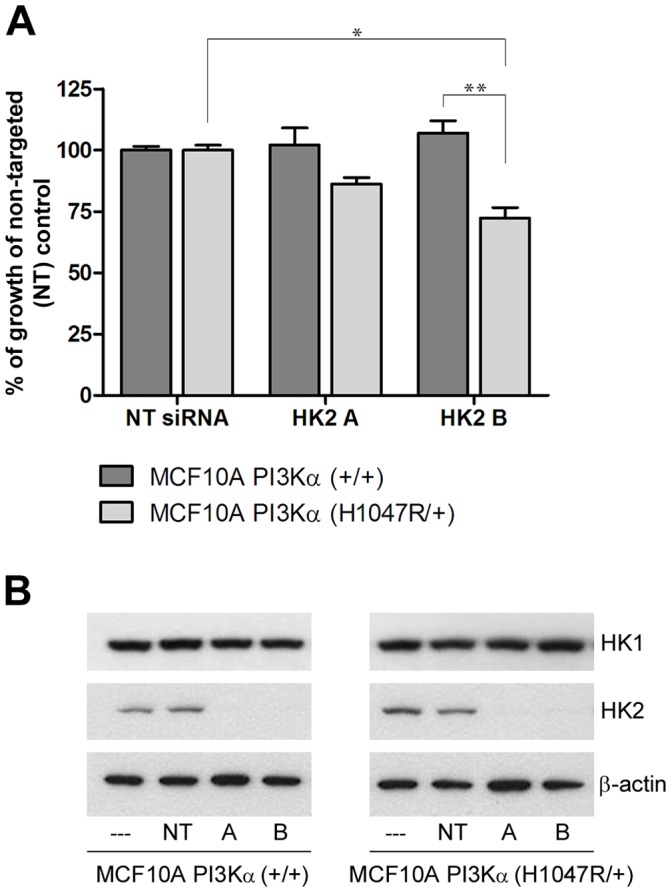
Targeted disruption of the glycolytic pathway demonstrates selective anti-proliferative effects in *PIK3CA* mutant cells. MCF10A PI3Kα (+/+) and MCF10A PI3Kα (H1047R/+) cells were transfected with non-targeted siRNA (NT) and 2 individual siRNA targeting *HK2*, HK2 A and HK2 B. (A) Cell growth was assessed 96 hours post-transfection using SRB. Growth was assessed as a percentage of non-targeted control (*p<0.05; **p<0.01). (B) Knockdown of HK2 protein was confirmed by Western blotting. HK1 Western blotting was performed to confirm specificity of knockdown.

**Table 1:** *PIK3CA* status of breast and colon cancer cell lines investigated for glucose dependency.

Cell Line	Cancer Type	*PIK3CA* mutation Status	Other genetic alterations
**RKO**	Colon	*H1047R*	*BRAF, NF1*
**MDA-MB-453**	Breast	*H1047R*	*CDH1*
**T47D**	Breast	*H1047R*	*p53*
**BT20**	Breast	*H1047R*+*P539R*	*p53, CDKN2*
**MCF7**	Breast	*E545K*	*CDKN2A*
**DLD-1**	Colon	*E545K*	*IDH1*

Additional genetic alterations are also shown.

**Figure 6:**
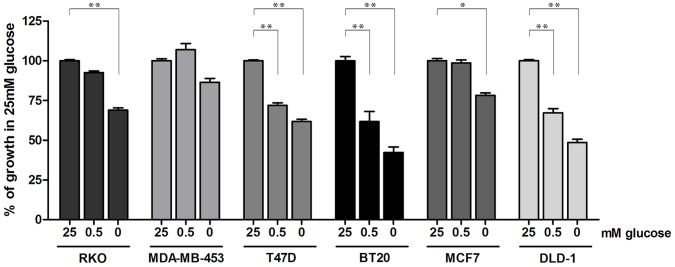
Glucose dependency is seen in multiple cancer cell lines harbouring endogenous *PIK3CA* mutations. Cell lines were grown for 120 hours in media containing 2 mM glutamine, and the indicated concentration of glucose and growth assessed using SRB. Growth of each cell line was expressed as a percentage of growth in 25 mM glucose (*p<0.05; **p<0.01).

### Growth Dependency Assays

Proliferation of cells over a 120 hour period was evaluated using the sulphorhodamine B (SRB) assay [35]. Cells were seeded into 96-well plates in triplicate in glucose and glutamine free DMEM (PAA) supplemented with the required concentration of glucose (Sigma) and glutamine (PAA). MCF10A isogenic cell lines were additionally supplemented with 5% horse serum, 10 µg/ml insulin, 0.5 µg/ml hydrocortisone, 0.1 µg/ml cholera toxin and 0.2 ng/ml hEGF. All other cell lines were supplemented with 10% foetal bovine serum only. Where indicated media was also supplemented with 0.5 mM fructose, 10 mM galactose, 0.5 ml/l fatty acid cell culture supplement or 0.1 mM aspartic acid (Sigma).

### Gene Expression Studies

MCF10A isogenic cell lines were seeded into 25 cm^2^ flasks in DMEM/F12 media supplemented with 5% horse serum, 10 µg/ml insulin, 0.5 µg/ml hydrocortisone, 0.1 µg/ml cholera toxin and 0.2 ng/ml hEGF. HCT116 isogenic cell lines were seeded into 25 cm^2^ flasks in McCoy’s 5A media supplemented with 10% foetal bovine serum. After 48 hours the cells were harvested by trypsinisation and RNA prepared using the RNeasy Kit (Qiagen), according to the manufacturer’s instructions.

Total RNA was reverse transcribed using the High Capacity cDNA Reverse Transcription Kit (Applied Biosystems). The transcription levels of 45 genes involved in glycolysis, glutaminolysis, pentose phosphate pathway, oxidative phosphorylation, and lipid metabolism were quantified by real-time PCR along with three normalization genes (*NUP214*, *PPIG* and *SLU7*). 5 ng of cDNA were used per PCR. Amplifications were performed with *ΔZO5* DNA polymerase in a buffer containing 20 mM Tris pH7.85, 30 mM KCl, 3 mM MgCl_2_, 100 µM dA,G,CTP, 200 µM dUTP, 0.8units uracil N-glycosylase, 6% glycerol, 1X ROX, and 0.2X SYBR green. The reactions were amplified on the Prism 7900 using the following cycling parameters: 50°C for 2 minutes, 95°C for 12 minutes, followed by 45 cycles of 95°C for 20 seconds and 60°C for 1 minute. A reference pool (Universal Human Reference RNA, Stratagene) at 2.5 ng was amplified with each gene. All amplifications were performed in duplicate.

Gene expression was determined using the 2(-ΔΔCt) method described by Livak and Schmittgen [36]. The expression of a gene in *PIK3CA* mutant cells relative to *PIK3CA* wild type cells was determined using the formula 2^(ΔΔCt PI3K mutated-ΔΔ Ct PI3K WT)^.

### Western Blotting

Cells were lysed in TG lysis buffer on ice and protein concentrations determined using the BCA protein assay (Sigma). Equal amounts of protein were loaded onto 10% Bis-Tris gels resolved by SDS-PAGE electrophoresis and transferred onto PVDF membrane (Invitrogen). Membranes were blocked in 5% w/v nonfat dry milk, 1x Tris-buffered saline, 0.1% Tween-20 for 1 hour at room temperature and then incubated in primary antibodies at 4°C overnight. The following antibodies were used: anti-GLUT1 (1∶5000 dilution; Epitomics 2944-1), anti-GLUT5 (1∶500 dilution; Santa Cruz ac-271055), anti-Hexokinase 1 (1∶500 dilution; Cell Signalling Technology 2804), anti-Hexokinase 2 (1∶500–1000 dilution; Cell Signalling Technology 2106), anti-MCT4 (1∶500; Millipore AB3314P), anti-phospho-AKT (1∶1000; Cell Signalling Technology 9271) and anti-β-actin (1∶500 000; Sigma A5441). Subsequently, membranes were incubated with the appropriate secondary antibody (Cell Signalling Technology) for 1 hour at room temperature. Proteins were detected using ECL-Plus (GE Healthcare) and exposure to film.

### siRNA Studies

ON-TARGETplus non-targeting pool siRNA (Dharmacon #D-001810-10-20) and two individual ON-TARGETplus siRNA against Hexokinase 2 (Dharmacon #J-006735-06 and #J-006735-07) were transfected into cells using Lipofectamine RNAiMAX (Invitrogen), following manufacturer’s instructions for forward transfection. Transfection complexes were left on cells for 6 hours before replacing with growth media. Cell growth was determined 96 hours post-transfection using the SRB assay as described above. Hexokinase 2 knockdown was confirmed by Western blotting as described, with Hexokinase 1 expression monitored to confirm specificity of knockdown.

### Statistical Analysis

Statistical analysis was performed using 2-way ANOVA tests followed by Bonferroni’s multiple comparison test. For simplicity, p values are represented as p<0.05 (*) or p<0.01 (**) only.

## Results

### The Presence of a PIK3CA Activating Mutation Results in Gene Expression Changes Associated with Increased Glycolytic Dependency and Additional Metabolic Pathway Alterations

To fully characterise the influence of a *PIK3CA* mutation on metabolic alterations as a whole, a targeted gene set was first compiled, representing 45 genes from different metabolic pathways such as glycolysis, oxidative phosphorylation, glutaminolysis, pentose phosphate pathway and fatty acid metabolism. Gene expression was then analysed across replicate RNA samples, prepared from two isogenic model systems. The first cell model system was a non-tumorigenic cancer cell line pair comprised of MCF10A parental cells in which PI3Kα is wild type (WT), PI3Kα (+/+) and MCF10A cells into which the *H1047R* activating mutation had been introduced, PI3Kα (H1047R/+). The second was a colon cancer cell line HCT116, which is heterozygous for the *H1047R PIK3CA* mutation, PI3Kα (H1047R/+), paired with cells in which the mutant allele has been knocked-out to render the cells functionally WT, PI3Kα (+/−). Analysis of gene expression for the *PIK3CA* mutant cell samples relative to *PIK3CA* WT cells revealed some striking alterations in metabolic gene expression levels. While the precise genes up- and down-regulated in the *PIK3CA* mutant cells compared to *PIK3CA* WT differed between the two cell model systems, the overall changes in gene expression were indicative of an increased aerobic glycolytic phenotype within *PIK3CA* mutant cells. Introduction of an activating mutation within *PIK3CA* in MCF10A cells led to an increase in expression of the key glycolytic enzyme *Hexokinase 2* (*HK2*), and a concurrent decrease in expression of the fructose transporter, *GLUT5* (Figure 1A). In the HCT116 colon cancer cell background, the presence of a mutated *PIK3CA* resulted in elevated expression of the glucose transporter, *GLUT1* (Figure 1B).

Other gene expression changes as a consequence of the presence of a *PIK3CA* mutation suggested that additional metabolic pathway alterations may exist. In MCF10A PI3Kα (H1047R/+) cells, the fatty acid transporter *SLC27A1* showed reduced expression, with several genes involved in oxidative phosphorylation also showing reduced expression (Figure 1A). Increases in the expression of genes such as *asparagine synthetase (ASNS)* and *glutaminase (GLS)* were consistently seen in PI3Kα (H1047R/+) cells, suggestive of elevated glutaminolysis (Figure 1A and 1B).

Changes in mRNA expression were then confirmed at the protein level, demonstrating elevated levels of key glycolytic enzymes such as HK2 and GLUT1, and additionally revealing up-regulation of the lactate transporter MCT4 in the MCF10A PI3Kα (H1047R/+) cells (Figure 2). Activation of the PI3K pathway was confirmed in both isogenic models as shown by AKT phosphorylation.

### Growth of PIK3CA Mutant Cells is Selectively Dependent on Glucose While Glutamine is Essential in Both WT and PIK3CA Mutant Cells

Results from the gene expression profiling were suggestive of a number of alterations in cellular metabolism as a result of mutated *PIK3CA*, specifically increased glycolysis and altered glutaminolysis. To explore the possibility that cells may be more dependent not only on specific pathways but also demonstrate nutrient source dependency, we investigated the effect of nutrient depletion on cellular growth, focusing on cellular dependency on glucose and glutamine. Over 120 hours, complete withdrawal of glucose (in the presence of glutamine) negatively impacted the ability of MCF10A PI3Kα (H1047R/+) to proliferate and grow, while having no effect on the growth of MCF10A PI3Kα (+/+) cells (Figure 3A). This differential impact was also true for a second MCF10A cell line containing an alternative *PIK3CA* mutation, MCF10A PI3Kα (E545K/+) (Figure 3A). Likewise, growth of HCT116 PI3Kα (H1047R/+) was more severely impacted following glucose depletion and withdrawal than HCT116 PI3Kα (+/−) cells (Figure 3B). This observation suggested that the *PIK3CA* WT cells were better able to tolerate glucose withdrawal than *PIK3CA* mutant cells, albeit in the presence of glutamine. To better understand the involvement of glutamine, we performed a matrix of studies, evaluating glucose depletion and withdrawal in conjunction with glutamine depletion and withdrawal. The growth of MCF10A cells, irrespective of *PIK3CA* mutation status was severely compromised by complete withdrawal of glutamine, even in the presence of glucose (Figure 3C). Partial depletion of glutamine also negatively impacted growth of the cells, but the extent of dependency did not appear to correlate to *PIK3CA* mutation status. Similarly, complete glutamine withdrawal severely compromised growth of HCT116 cells. In contrast to MCF10A cells, HCT116 cells were better able to tolerate an intermediate level of glutamine withdrawal, with no growth impairment seen in either *PIK3CA* WT or mutant cells (Figure 3D). The pattern of glucose sensitivity was maintained with this intermediate level of glutamine (Figure S1).

### The Glucose Dependency Exhibited by PIK3CA Mutant Cells Cannot be Overridden by Supplementation with Other Nutrients

In order to further characterise the glycolytic phenotype and apparent glucose dependency of *PIK3CA* mutant cells, we used the MCF10A isogenic model system to evaluate whether alternative nutrient sources could compensate for glucose and override the dependency. Switching the nutrient source to an alternative monosaccharide (fructose or galactose) was unable to compensate for glucose withdrawal in MCF10A PI3Kα (H1047R/+) cells, while growth of MCF10A PI3Kα (+/+) cells remained unaffected by removal of glucose or nutrient switching (Figure 4). Since gene expression data suggested that *PIK3CA* mutant cells may have alterations in metabolic pathways other than glycolysis, we further investigated whether the glucose growth dependency could be overridden by supplementing with nutrients that may promote these alternative pathways. Based on gene expression changes in asparagine synthetase and fatty acid transporters, we supplemented cells with aspartic acid or a mixture of fatty acids. Neither supplement was able to override the effects of withdrawing glucose on the growth of MCF10A PI3Kα (H1047R/+) cells (Figure 4). Similar results were seen when the MCF10A PI3Kα (E545K/+) and HCT116 PI3Kα (H1047R/+) cells were profiled (Figure S2).

### Disruption of the Glycolytic Pathway Demonstrates Anti-proliferative Effects in PIK3CA Mutant Cells

The MCF10A PI3Kα (H1047R/+) cells show a high level of glucose dependency and are unable to compensate for the absence of glucose by utilizing other nutrients. In conjunction with our data demonstrating that *PIK3CA* mutant cells show increased expression of the key glycolysis enzyme HK2, we hypothesised that disruption of the glycolytic pathway should be sufficient, even in the presence of glucose, to inhibit growth of the mutant cells. We therefore evaluated the effects of targeted disruption of glycolysis using two siRNA molecules to knockdown levels of HK2 in the MCF10A isogenic cells. Using a non-targeted siRNA molecule as a control, we found that specific HK2 knockdown does indeed result in anti-proliferative effects, selective for the *PIK3CA* mutant cells (Figure 5A). Knockdown of HK2 protein levels in both *PIK3CA* WT and mutant cells lines was confirmed by Western blotting (Figure 5B), with specificity of knockdown confirmed by monitoring HK1 expression.

### Glucose Dependency is Seen in Other Cancer Cell Lines Harbouring Endogenous PIK3CA Mutations

Our investigations have used a precise and genetically defined system to investigate the effects of a *PIK3CA* mutation on cellular metabolism in isolation. We extended our studies to understand whether these findings were true across a broader panel of cancer cell lines. We therefore determined the glucose growth dependency of six colon and breast cancer cell lines, all containing endogenous *PIK3CA* mutations (Table 1). However, these lines also have the caveat of having a number of other genetic differences, which may modulate or bypass the putative glucose dependency determined in isogenic cell-systems. The extent to which growth was affected varied between the lines, with 3 of the 6 cell lines (T47D, BT20 and DLD-1) demonstrating reduced growth following partial glucose depletion (Figure 6). However, growth of all 6 cell lines was compromised by complete withdrawal of glucose even in the presence of glutamine, supporting the concept that *PIK3CA* mutant cancers are heavily reliant on glucose to support cell growth.

## Discussion

Interest in studying cancer metabolism and developing metabolic targeted agents as therapies has grown over recent years, and so an improved understanding of the unique metabolism of cancer cells is imperative for effective drug design and the rational treatment of patients, as well as labelled nutrient visualisation and treatment response monitoring. Despite activation of the PI3K-AKT-mTOR pathway being a common feature of many cancers, and activation of this pathway being associated with regulation of a number of metabolic effects, comprehensive profiling of the consequence of *PIK3CA* mutations on metabolic dependencies has not been studied. By using genetically defined isogenic cell models, we have been able to precisely define how *PIK3CA* mutations can influence metabolic pathways within a tumour.

Regulation of metabolic processes and nutrient utilisation are complex processes. Gene expression analysis in this study has revealed that the overall consequence of *PIK3CA* mutations is a shift towards an increased glycolytic phenotype. However, the exact way a cell effects this shift may vary. Rather than a single rate limiting molecule, several steps creating a ‘regulatory pathway’ may share the homeostatic control of energy metabolism. Multi-step control was highlighted by the differential gene up-regulation of key glycolysis enzymes in the different isogenic models. Though differences were seen, the net effect is suggestive of up-regulation of glycolysis in both cases. This link between patient relevant *PIK3CA* mutations and resulting glycolytic phenotype is consistent with previous studies that have associated activation of the AKT signalling pathway with stimulating the Warburg effect [11], [13]. This concept is built on and strengthened by having revealed a clear dependence of *PIK3CA* mutant cells on glucose for continued growth. Despite suggestions that cancer cells are adept at utilizing other nutrient sources, this study has demonstrated that *PIK3CA* mutant cells are not readily able to compensate for glucose withdrawal, either through use of glutamine, alternative monosaccharides or other nutrients. Dependence on glucose for growth was also shown across a wider panel of cancer cell lines harbouring endogenous *PIK3CA* mutations. The results of this study could provide critical information for designing therapeutic strategies, since they have highlighted critical nodes within metabolic pathways that might be key to target. Indeed, knockdown studies confirmed that targeting the glycolytic pathway for disruption results in anti-proliferative effects, specific to *PIK3CA* mutant cells, suggesting that this could be a therapeutic strategy for specifically targeting patients with *PIK3CA* mutant tumours. The overt glucose dependence of cells with this mutation also offers routes for non-invasive imaging of PI3K-pathway activated tumours.

Through characterisation of the metabolic phenotype of genetically matched isogenic cells, we have been able to define the metabolic consequences of a single engineered genetic alteration, in this case an activating mutation within the *PIK3CA* gene. Whilst confirming that activation of the PI3K-AKT-mTOR pathway results in an increased glycolytic phenotype, we have also demonstrated a specific dependence for *PIK3CA* mutated cells on glucose and illustrated that targeting this pathway offers therapeutic potential and tumour imaging insight.

## Supporting Information

Figure S1 **Glucose sensitivity of HCT116 PI3Kα (H1047R/+) cells was maintained when grown in 0.5 mM glutamine media.** HCT116 isogenic cells (+/− and H1047R/+) were grown for 120 hours in media containing 0.5****mM glutamine and the indicated concentrations of glucose. Cell growth was assessed using SRB staining. The growth of each cell line is expressed relative to growth in media containing 0.5****mM glutamine and 25****mM glucose.
(TIF)

Figure S2 **The glucose dependency of** ***PIK3CA*** **mutant cells cannot be overridden by supplementation with alternative nutrients.** MCF10A PI3Kα isogenic cells (+/+ and E545K/+) and HCT116 isogenic cells (+/− and H1047R/+) were grown for 120 hours in media containing 2****mM glutamine and the indicated concentrations of glucose (A and B respectively). Additionally, cells grown without glucose were supplemented with either fatty acid cell culture supplement (+FA) or 0.1****mM aspartic acid (+AA). Cell growth was assessed by SRB staining.
(TIF)
