# Supplementary figures and images for: Multiple Metabolic Alterations Exist in Mutant PI3K Cancers, but Only Glucose Is Essential as a Nutrient Source
Source: PLoS One. 2012 Sep 13;7(9):e45061. doi: 10.1371/journal.pone.0045061 (PMC3441563; doi:10.1371/journal.pone.0045061)

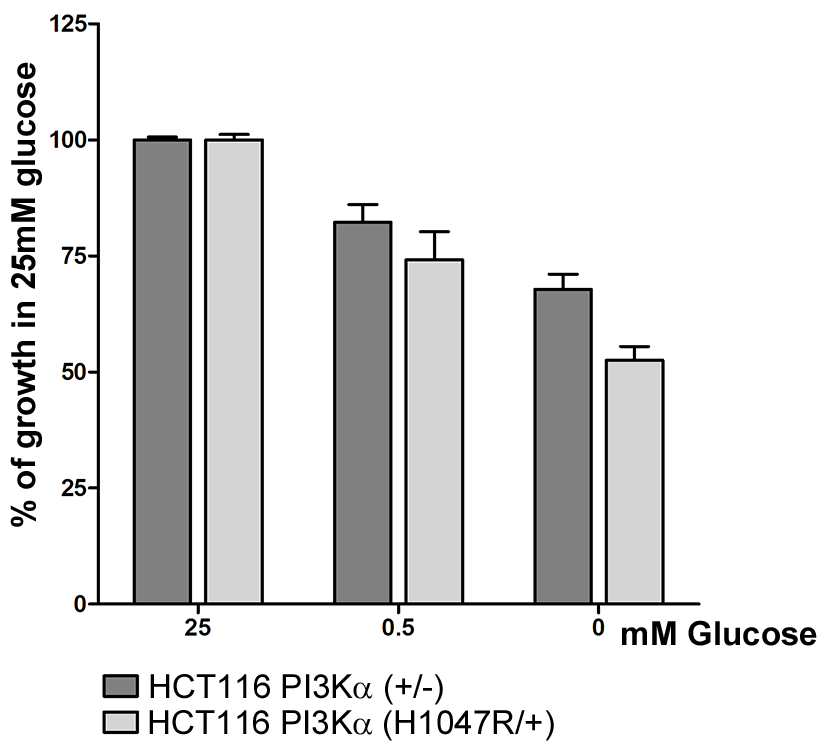

Supplement: Figure S1 — Glucose sensitivity of HCT116 PI3Kα (H1047R/+) cells was maintained when grown in 0.5 mM glutamine media. HCT116 isogenic cells (+/− and H1047R/+) were grown for 120 hours in media containing 0.5mM glutamine and the indicated concentrations of glucose. Cell growth was assessed using SRB staining. The growth of each cell line is expressed relative to growth in media containing 0.5mM glutamine and 25mM glucose. (TIF) [file pone.0045061.s001.tif]

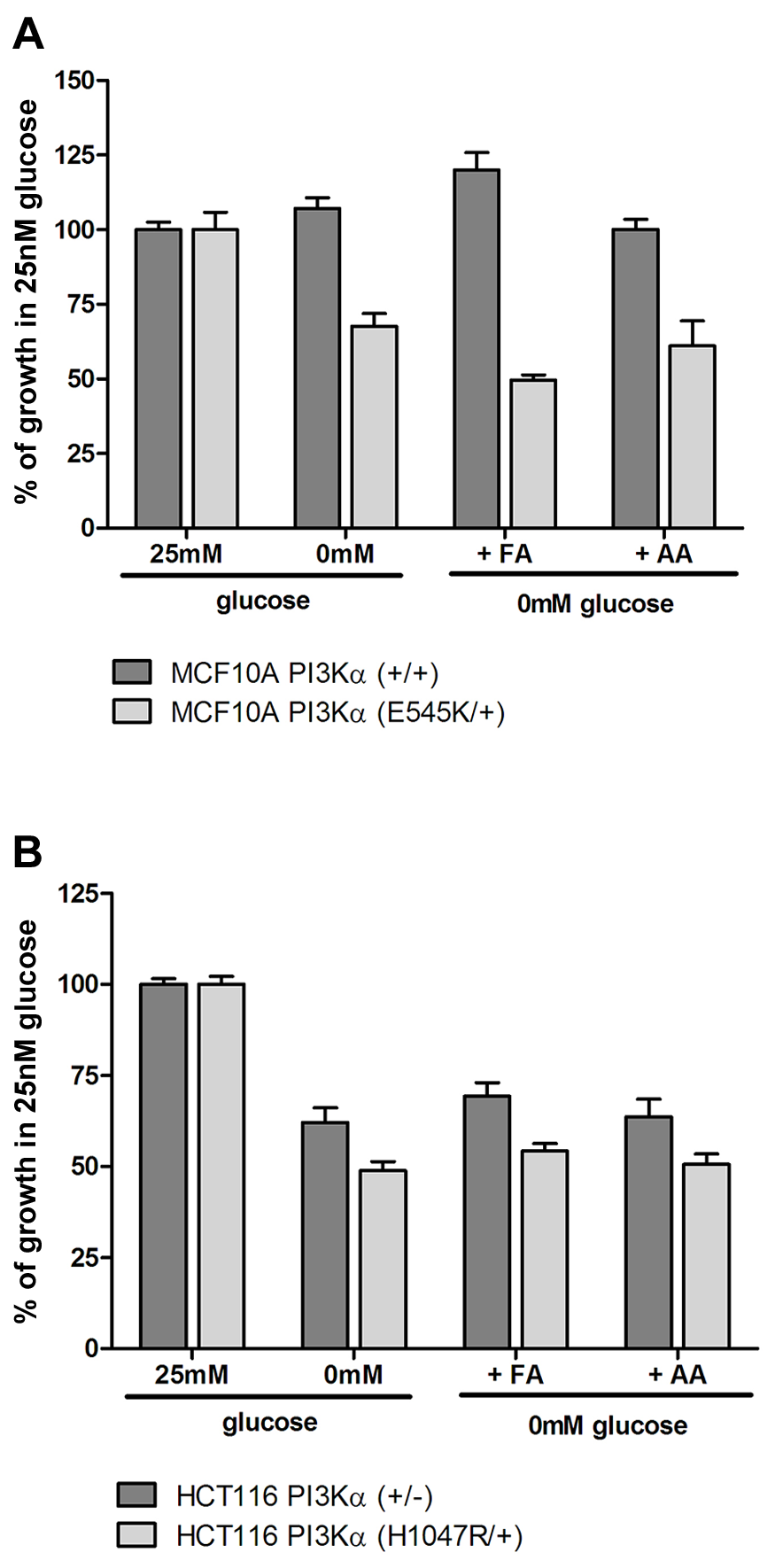

Supplement: Figure S2 — The glucose dependency of PIK3CA mutant cells cannot be overridden by supplementation with alternative nutrients. MCF10A PI3Kα isogenic cells (+/+ and E545K/+) and HCT116 isogenic cells (+/− and H1047R/+) were grown for 120 hours in media containing 2mM glutamine and the indicated concentrations of glucose (A and B respectively). Additionally, cells grown without glucose were supplemented with either fatty acid cell culture supplement (+FA) or 0.1mM aspartic acid (+AA). Cell growth was assessed by SRB staining. (TIF) [file pone.0045061.s002.tif]
